# Characterization of a Collection of Natural Oleogenic Yeasts to Identify Promising Producers of Food Oil Analogues

**DOI:** 10.3390/ijms27020578

**Published:** 2026-01-06

**Authors:** Igor A. Cherdantsev, Alexandra N. Poliakova, Artemiy S. Silantyev, Viktoriia D. Kazakova, Alexandra D. Samojlova, Nikita B. Polyakov, Polina D. Belkina, Arina A. Simonova, Alina S. Bogdanova, Anna V. Kudryavtseva, Natalya S. Gladysh, Dmitry S. Karpov

**Affiliations:** 1 Engelhardt Institute of Molecular Biology, Russian Academy of Sciences, Moscow 119991, Russia; igor.cherdancev.2012@mail.ru (I.A.C.); polyakovaan@my.msu.ru (A.N.P.); arinasimonova725@gmail.com (A.A.S.); alina.bogdashka@yandex.ru (A.S.B.); rhizamoeba@mail.ru (A.V.K.); natalyagladish@gmail.com (N.S.G.); 2 Department of Soil Biology, Faculty of Soil Science, M.V. Lomonosov Moscow State University, Leninskie Gory, 1, Moscow 119234, Russia; ribaluna2003@gmail.com (A.D.S.); pb3lkina@yandex.ru (P.D.B.); 3 Center for Molecular and Cellular Biology, Moscow 121205, Russia; 4 Lopukhin Federal Research and Clinical Center of Physical-Chemical Medicine of Federal Medical Biological Agency, Moscow 143007, Russia; artsilan@gmail.com (A.S.S.); viktorialuzanova.1998@gmail.com (V.D.K.); 5 Gamaleya National Research Centre for Epidemiology and Microbiology, Ministry of Health of the Russian Federation, Moscow 123098, Russia; 6 Vernadsky Institute of Geochemistry and Analytical Chemistry, Russian Academy of Sciences, Moscow 119334, Russia; 7 Faculty of Biology and Biotechnology, National Research University Higher School of Economics, Moscow 101000, Russia

**Keywords:** oleaginous yeasts, fatty acids, triacylglycerols, carbon source, food oil

## Abstract

The production of fats and oils represents a task that is in demand in a variety of industries, including the food industry. Presently, the predominant method of acquiring them is through the processing of plant and animal products, a process that substantially increases the cost of production at all stages. Oleaginous yeasts can serve as an alternative source for obtaining edible oils, as demonstrated by yeast strains employed in the development of palm oil analogues. In this study, we created and characterized a collection of oil-producing yeast species obtained from various natural sources. These species were identified using MALDI-TOF and Sanger sequencing. The isolates were qualitatively and quantitatively tested for their ability to grow on various culture media compositions. The oil-producing strains were characterized by their fatty acid profile and lipidome composition. In addition, we evaluated the biotechnological potential of these organisms as producers of fatty acid- and fat-related products. As a result, the collection contains 100 strains, 31 of which are oleaginous yeasts, and three strains show potential as promising producers of edible oil analogues. Our research demonstrates the benefits of searching for and studying natural yeast strains, both from a fundamental science perspective and for the creation of future innovative biotechnological solutions in the food industry.

## 1. Introduction

Fats and oils are essential components of human and animal nutrition [1] and are significantly involved in the production of food industry products [2]. The main sources of these products are plant crops (oils) and livestock (fats). According to analytical reports, the global market for oils and fats will grow by 3–5% over the next five years, due to rapid economic growth and increasing consumer interest in healthier eating [3,4,5]). However, this market is characterized by high volatility due to changes in acreage and yields [6], which in turn leads to higher product prices. These changes are not least due to climate change, which not only leads to increased costs [7,8], but can also reduce product quality [9].

There is no doubt that the need to increase the production of this type of product will grow. However, the sector faces a number of critical challenges. Significant growth in global demand for vegetable oil is forecast, with production needing to increase by 74% by 2050 to meet the food needs of an expected 9.2 billion people [10]. This growth will require approximately 317 million hectares of additional land for oilseed cultivation, which will lead to a significant increase in acreage and, as a result, to higher raw material prices [11]. The growing market demand for vegetable oils is driven by a number of factors: increased food consumption in developing countries, growing demand for biodiesel and environmentally friendly fuels, as well as their use in cosmetics, pharmaceuticals, and industrial lubricants [12]. These market characteristics necessitate the search for alternative sources of oils.

However, several problems must be solved at once in order to create a successful technological solution. First, fats and oils are multicomponent substances that include not one but several fractions of compounds [13], represented mainly by triglycerides, saturated, mono- and polyunsaturated fatty acids [1]. Second, the alternative approach should be significantly cheaper than traditional production methods and should be less dependent on climate and other natural conditions that may be associated with product instability. A promising solution that meets all these conditions could be the cultivation of microorganisms that synthesize and accumulate large amounts of lipids [14].

The fundamental advantage of microbial lipid production is that oil-producing microorganisms are capable of accumulating lipids in quantities exceeding 20% of their dry cell weight, and some fungi are capable of reaching 70–80% lipid content of their total dry weight [15]. These microorganisms are capable of producing a fatty acid composition comparable to or superior to that of traditional vegetable oils, including essential polyunsaturated fatty acids (PUFAs) [16]. Various groups of organisms can be used as producers of oil analogues: microalgae [17], bacteria [18], yeasts and fungi [19,20]. Each group of producers has a number of advantages and disadvantages: for example, microalgae are characterized by high nutritional value, but their cultivation is associated with a number of difficulties that require specialized technological developments [21,22]. Bacteria have not received as much attention as lipid producers. In this regard, the use of yeast and fungi seems more justified, since industrial technologies have been developed for their cultivation, and industrial strains are well studied from a biotechnology perspective.

Undoubtedly, there are several significant advantages to considering oleogenic yeasts and fungi. Among them, we can highlight the possibility of using non-food substrates as a carbon source [23], independence from climatic conditions and the agricultural sector [24], and a relatively short production cycle [25]. At the same time, there are serious limitations that prevent laboratory developments from entering the commercial sector [25]. First and foremost, the high cost of production remains problematic, due to the need to select a substrate composition that will yield the optimal amount of lipids [26]; this necessity leads to the inevitable use of high-quality sources, which runs counter to the need to reduce the cost of the production cycle. Another problem hindering the commercialization of production is the complexity of scaling technologies [25,27].

Among the 1600 species of yeast described, about 10% are considered oleogenic, i.e., capable of synthesizing and accumulating lipids and triacylglycerides (TG) inside the cell [28,29], in quantities exceeding 20% of biomass [30]. Among this multitude of species, there are strains that have proven themselves well as producers of oils. Among oleogenic yeasts, strains of *Yarrowia lipolytica* [31] are often used: for example, it was recently used to develop an experimental approach to obtaining a breast milk fat substitute [32], and even earlier, a strain was developed to increase the accumulation of beta-carotene [33] and the omega-3 fatty acids eicosapentaenoic acid [34]. There are several other species that are emerging as promising oil producents, e.g., *Rhodotorula toruloides*, *Cutaneotrichosporon oleaginosus*, *Lipomyces starkeyi*, and *Rhodotorula glutinis*, all of which differ in both their fatty acid content and the amount they accumulate [29,35]. These differences, on the one hand, limit the use of species as producers, but on the other hand, allow for the selection of more suitable strains for development.

These differences in the metabolic characteristics of oleogenic yeasts make it possible to search for and select wild types or traditional strains that exhibit a lipid composition close to that of the target product. The isolation of oleogenic strains from natural sources and their screening is one of the most important and fundamental stages in the development of new biotechnological solutions, creating a platform for the selection of a potential producer [36], and through wise selection, it becomes possible to optimize the time and financial costs of developing metabolic and genetic engineering approaches. If a promising producer is found, it becomes possible to select cultivation conditions that solve at least one problem, namely the high cost of the cultivation cycle. For example, it is possible to find microorganisms that can highly efficiently use inexpensive components, such as industrial waste, as a substrate.

To achieve this, two fundamental tasks must be solved: first, to identify strains capable of accumulating large amounts of lipids, and second, to develop an approach that will facilitate the reliable identification of promising candidates for industrial use. Such screening is aimed precisely at selecting strains that can be cultivated at a relatively low cost. The aim of this work is to search for new promising strains of oleogenic yeast among the strains inhabiting the surfaces of fruits and vegetables. Yeast isolates obtained from the surface of fruits were identified using MALDI-TOF and ITS sequencing. Oleogenic strains were selected using a glycerol flotation test. Next, the ability of the isolated strains to assimilate various carbon sources, including inexpensive organic waste from the food industry, was determined. The profile of major fatty acids in the oil isolated from the most productive strains was determined using GC-MS, and the composition of major triglycerides was determined using LC-MS. Our data allowed us to identify three natural oleogenic strains that could serve as potential producers of edible oils.

Thus, this work represents a large-scale study of yeast, which describes an effective approach for evaluating strains that have high potential to become a platform for the biotechnological production of vegetable oil analogues and their individual components.

## 2. Results

### 2.1. Identification of Isolates in Yeast Collection

**Figure 1:**
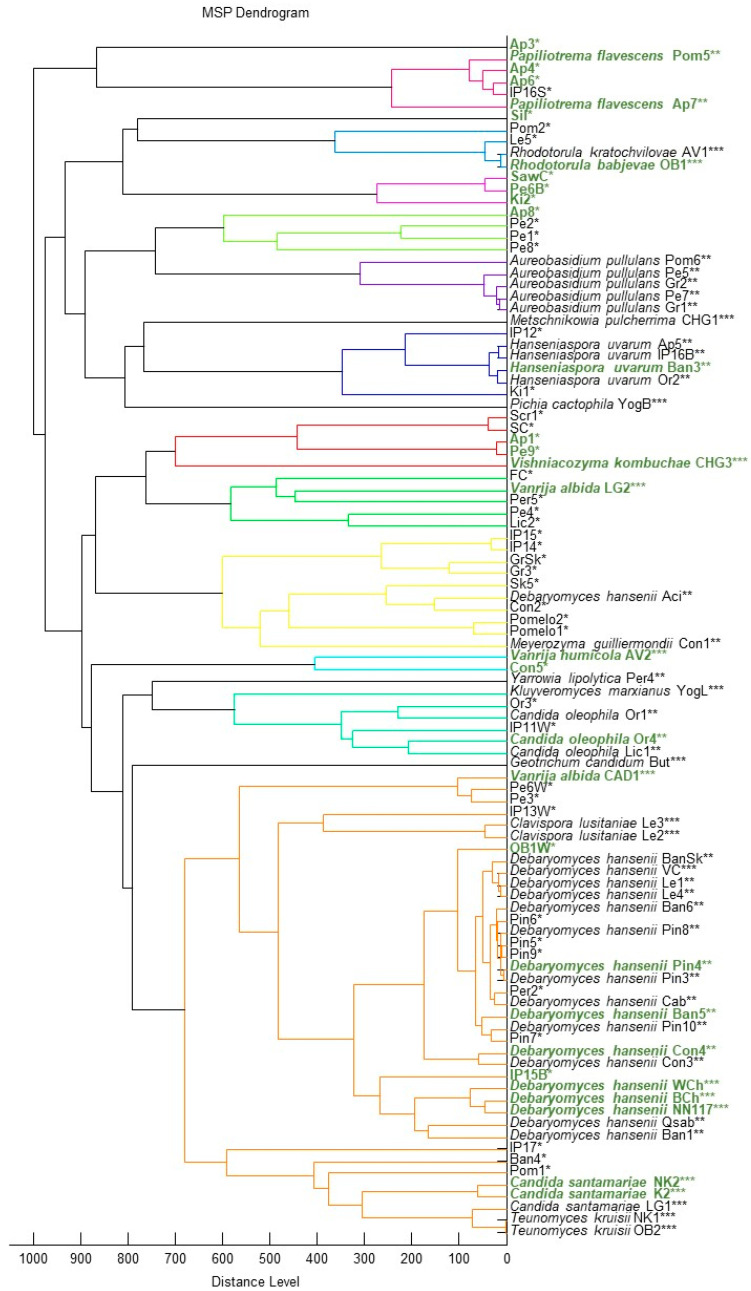
Dendrogram based on MALDI-TOF data of yeast strains obtained during the search for oleogenic strains with biotechnological potential. Star symbols: ***—secure genus identification, probable species identification (range 2.000–2.299); **—probable genus identification (range 1.700–1.999); *—not reliable identification (0.000–1.699). Oleogenic strains that passed the 20% glycerol flotation test are highlighted in green.

During the search for oleogenic yeasts, 100 pure cultures of yeast isolates were isolated and their primary identification was performed using MALDI-TOF (Figure 1).

According to MALDI-TOF data, out of 100 isolates analyzed, 52 were identified with sufficient reliability to the genus level, and 15 of them to the species level.

**Figure 2:**
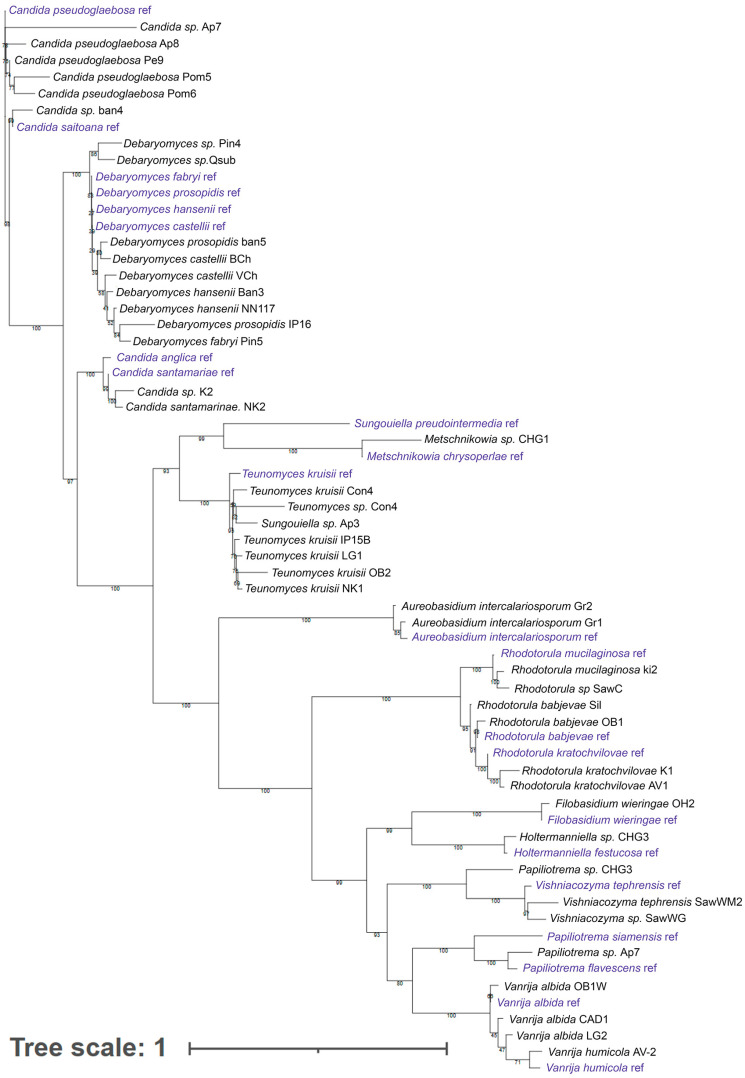
Dendrogram constructed based on ITS region sequencing data for strains representing all clades identified by MALDI-TOF analysis.

Identification using MALDI-TOF of fungal isolates from natural sources and food products, in general, can be difficult [37,38,39] due to the limited size of the microorganism database. In this regard, strains representing clades identified using MALDI-TOF were identified by sequencing the ITS1-5.8S-ITS2 regions. The results are shown in Figure 2 (the list of sequences is provided in Table S1).

### 2.2. Physiological Characteristics for Oleogenic Strains

**Figure 3:**
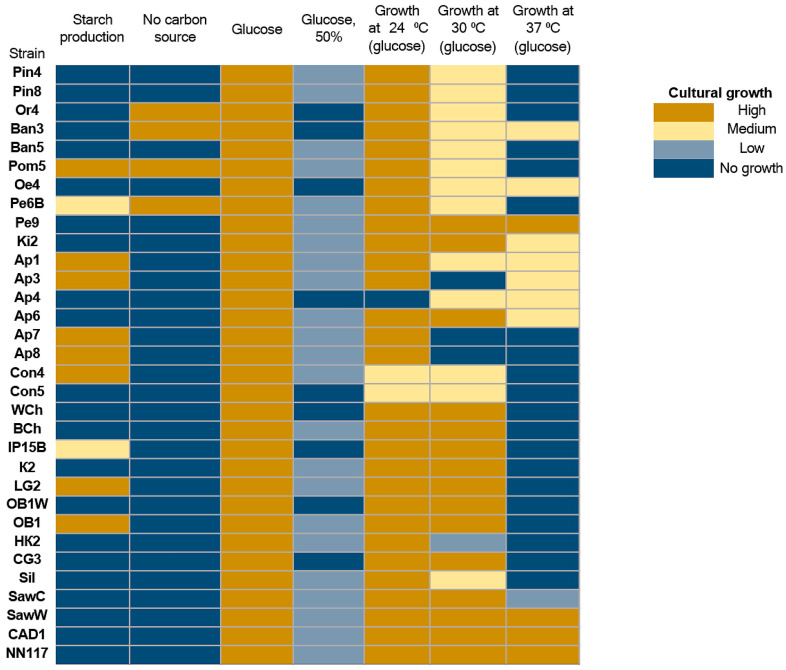
Evaluation of the ability of oleogenic strains to grow on nutrient media without a carbon source and with the addition of glucose as a carbon source. under hyperosmotic conditions (50% glucose) and under various (or without a carbon source) as a carbon source under varying cultivation conditions. No growth—no growth, Low—single colonies, Medium—continuous biomass growth, but significantly less than in the positive control, and High (+)—biomass growth comparable to the positive control or more abundant.

Yeast isolates were also characterized for oleogenicity using a flotation test in 20% glycerol (Figure 1, marked in green, Table S2, marked in yellow), as well as for their ability to grow on media with different carbon sources (Table S2). A total of 31 oleogenic strains were detected. In the case of oleogenic strains, the test results show the ability of some strains to grow in the absence of a carbon source in the culture medium: Or4, Ban3, Pom5, Pe6B (Figure 3). The ability of these yeast strains to grow in a carbon-free environment indicates that they are capable of effectively fixing CO_2_ from the air. Several enzymes capable of fixing CO_2_ have been described, such as pyruvate carboxylase, phosphoenolpyruvate carboxylase, and acetyl-CoA carboxylase [40,41]. However, there are no explicit references in the literature to natural yeasts capable of growing on media without carbon sources. It can be assumed that the strains we have discovered possess more active enzymes, such as acetyl-CoA carboxylase, which allow them to effectively fix CO_2_. It is also possible that the strains we have described have a symbiotic relationship with bacteria capable of effectively fixing CO_2_.

**Figure 4:**
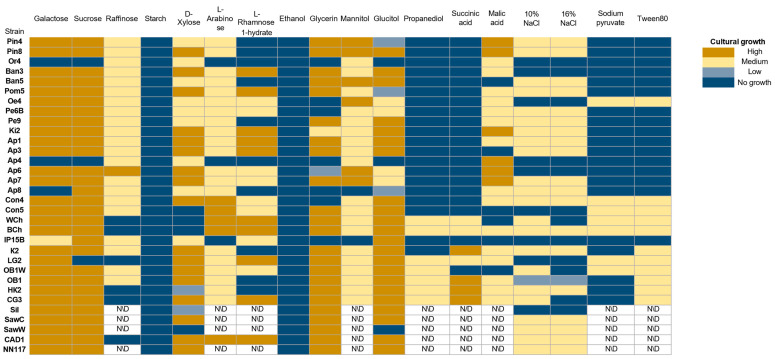
Evaluation of the ability of oleogenic strains to grow on various carbon sources. No growth—no growth, Low—single colonies, Medium—continuous biomass growth, but significantly less than in the positive control, and High (+)—biomass growth comparable to the positive control or more abundant. N/D—not determined.

All strains are capable of growing in a nutrient medium with a standard amount of glucose. However, high glucose concentrations (50%), which create hyperosmotic conditions, inhibit the growth of all strains studied. Most oleogenic strains retain their ability to grow at relatively low temperatures, and only a few are capable of growing at temperatures above 30 °C (Pe9, CAD1, and NN117). In general, oleogenic strains differ significantly in their physiological abilities, including their ability to metabolize various carbon sources (Figure 4).

Almost all strains demonstrate the ability to grow on media containing galactose, sucrose, D-xylose, glycerol, and sorbitol. The use of other compounds (raffinose, L-arabinose, mannitol, malic acid) as a carbon source leads to a significant decrease in the rate of biomass accumulation. All strains studied are unable to metabolize starch and ethanol. Starch-degrading strains are mainly described in the ascomycete genera *Endomycopsis*, *Lipomyces*, *Candida*, *Pichia*, *Debaryomyces*, and *Schwanniomyces* [42,43]. *Rhodotorula* strains capable of hydrolyzing starch are also known [44]. In addition, all strains show growth repression under hyperosmotic conditions caused by increased NaCl concentration, which is consistent with the sensitivity of the studied strains to hyperosmotic conditions caused by increased glucose concentration (Figure 3).

**Figure 5:**
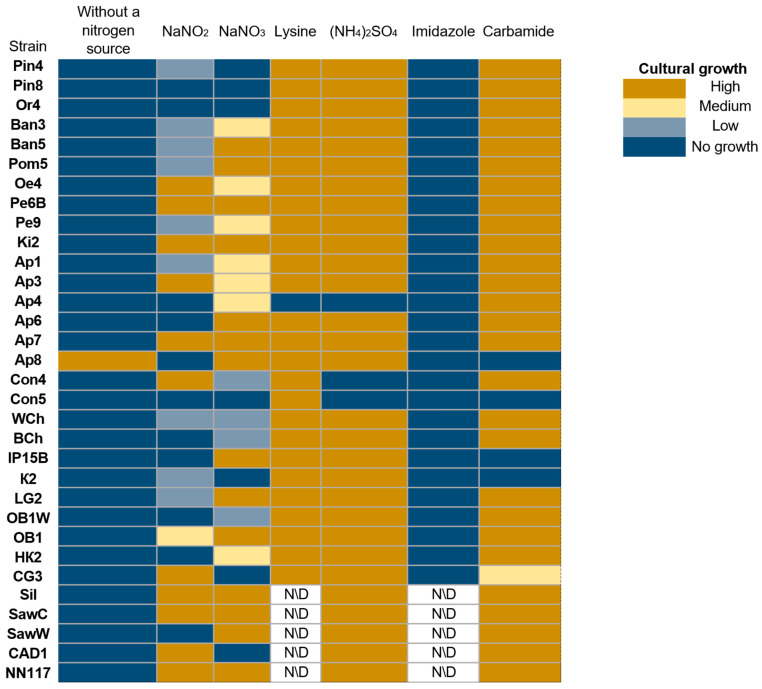
Evaluation of the ability of oleogenic strains to utilize different nitrogen sources. No growth—no growth, Low—single colonies, Medium—continuous biomass growth, but significantly less than in the positive control, and High (+)—biomass growth comparable to the positive control or more abundant.

The type of nitrogen source and the ability to metabolize it determine the ability of yeast strains to form biomass [45,46]. As a next step, we evaluated the ability of strains to grow on different nitrogen sources (Figure 5).

Interestingly, one of the oleogenic strains, Ap8, demonstrated the ability to grow on a nutrient medium without a nitrogen source. However, no nitrogen-fixing yeasts have been described in the literature. There is information that *Rhodotorula mucilaginosa* strains have nitrogen-fixing endosymbiotic bacteria *Bacillus velezensis*, *Staphylococcus stutzeri*, *Lysinibacillus telephonicus*., *Brevibacillus* sp., and *Niallia circulans* [47]. The possibility of co-cultivation of yeasts, for example, *Yarrowia lipolytica* and the nitrogen-fixing bacterium *Azotobacter vinelandii*, has also been demonstrated [48]. Of the 31 strains, only 9 are capable of assimilating NaNO_2_, and 13 strains are capable of assimilating NaNO_3_. Most strains, with the exception of Ap4, are capable of assimilating lysine and ammonium sulfate, which are readily available sources of nitrogen for yeast [49,50]. Most strains are also capable of assimilating urea. At the same time, none of the tested strains are capable of assimilating imidazole.

### 2.3. Characterization of Growth and Fatty Acid Profile of Oleogenic Strains on Media with Glucose as a Carbon Source

**Figure 6:**
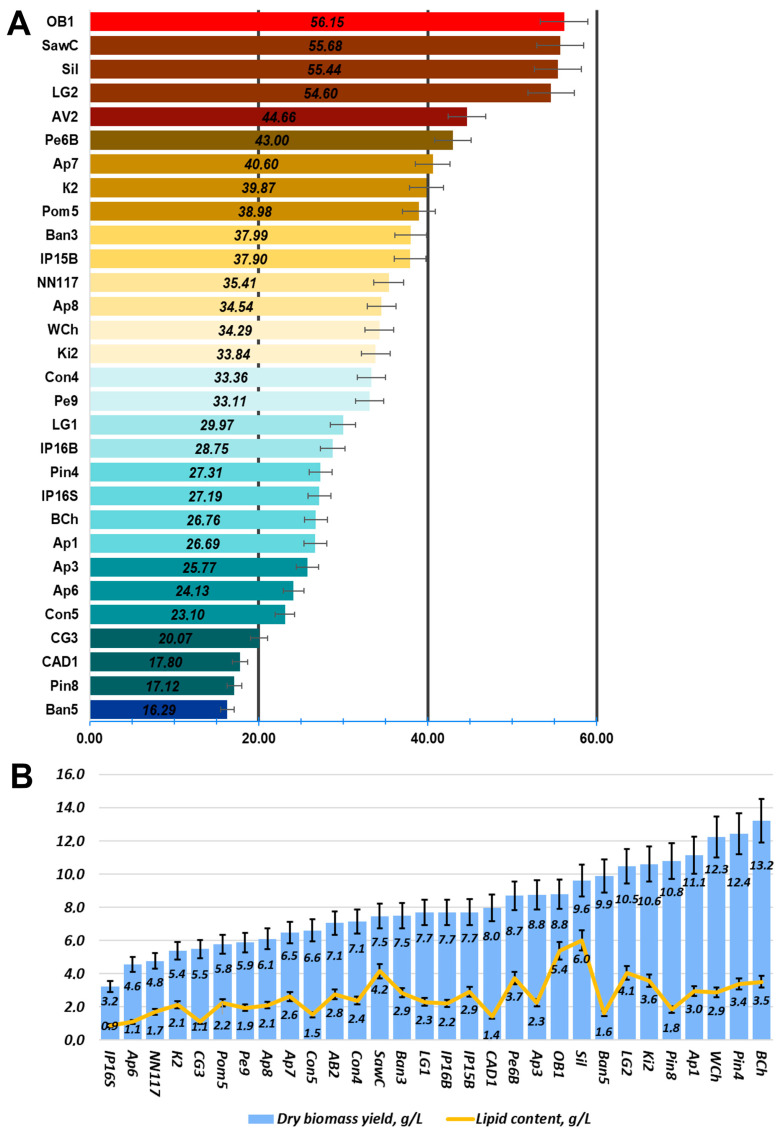
Productivity of the studied oleogenic strains when grown on oleogenic medium A with glucose as a carbon source. (**A**) Oil content of oleogenic strains. Total oil per dry biomass. (**B**) Dry biomass and total oil yield of oleogenic strains.

Oleogenic strains were characterized for oil content, dry biomass, and oil yield when cultured on medium A using glucose as a carbon source. The data are presented in Figure 6.

**Figure 7:**
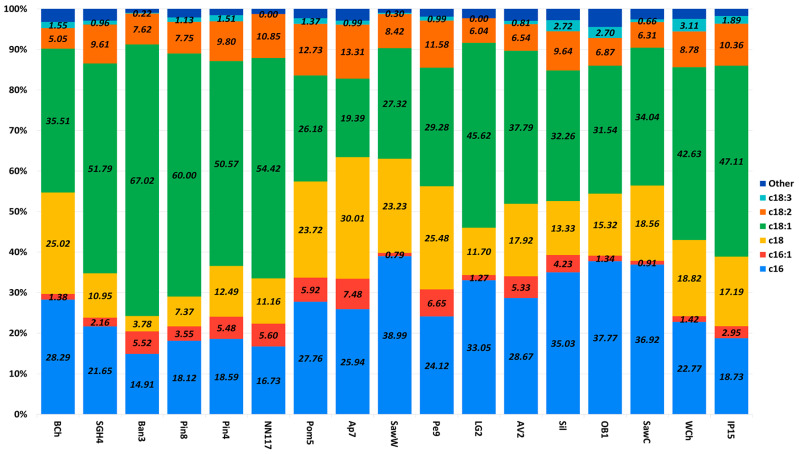
Fatty acid profile in oleogenic strains of interest. The data are presented for cultures grown on medium A with glucose as a carbon source. A statistical analysis of the data is provided in Table S3.

Based on the oil content data (Figure 6), the most promising strains from the collection were selected. Their fatty acid profiles were analyzed when cultivated on standard oleogenic medium A using glucose as a carbon source (Figure 7).

According to the data obtained (Figure 7), the highest content of saturated fatty acids (palmitic and stearic), reaching a value of 70%, is observed in strain Ap7. The highest content of unsaturated fatty acids (oleic and linoleic), reaching a value of almost 75%, is observed in strain Ban7. In terms of the content of individual fatty acids, the oil isolated from strain Ban7 is enriched with oleic acid (67%), linoleic acid is most abundant in oil from strain Pom5 (~13%), the highest content of stearic acid is observed in strain Ap7, and the highest content of palmitic acid is observed in strain SawW (39%).

### 2.4. Characterization of Growth and Fatty Acid Profile of Oleogenic Strains on Alternative Carbon Sources

**Figure 8:**
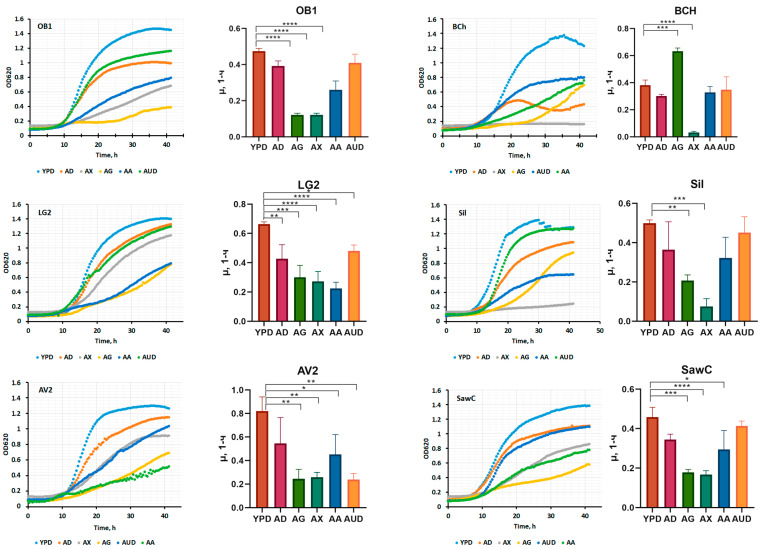
Biomass accumulation kinetics data for the best strains. Culture media, abbreviations: YPD—yeast peptone dextrose, AD—medium A with glucose, AG—medium A with glycerol, AX—medium A with xylose, AUD—medium A with urea instead of ammonium sulfate, and AA—medium A with potassium acetate. the *p* value is adjusted: ****—<0.0001; ***—0.0001 < *p* <0.001; **—0.001 < *p* < 0.01; *—0.01 < *p* < 0.1.

The best oleogenic strains showing the highest yields of oil and dry biomass were used in further experiments to determine the growth kinetics on various carbon sources (Figure 8).

**Figure 9:**
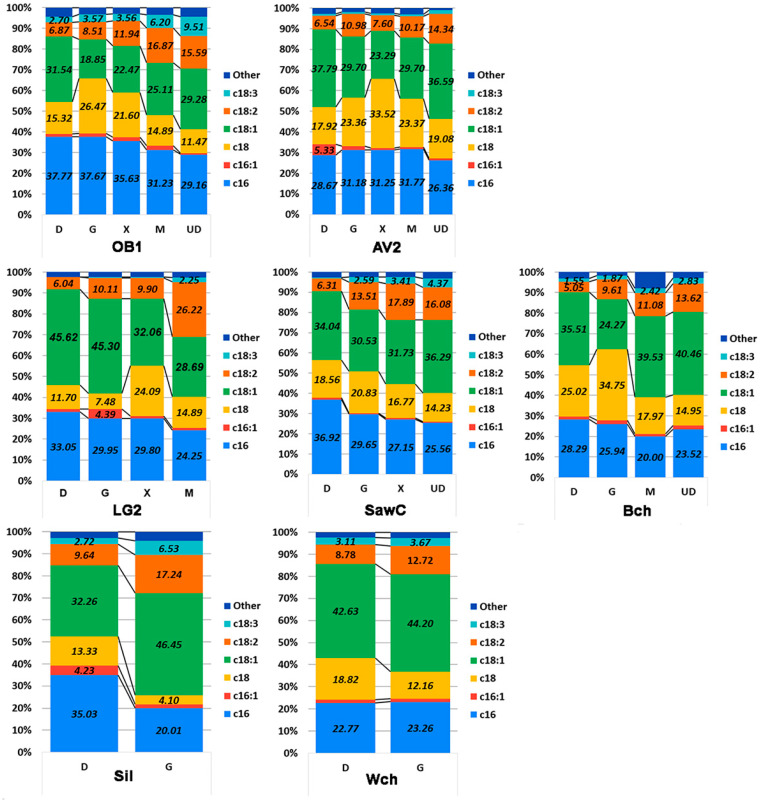
Profile of the content of the main fatty acids in the fattiest oleogenic strains when grown on an oleogenic medium with different sources of carbon and nitrogen. Designations: D—glucose, G—glycerol, X—xylose, M—beet molasses, and UD—glucose as a carbon source, and urea as a nitrogen source instead of ammonium sulfate. A statistical analysis of the data is provided in Table S4.

Since the carbon source strongly influences the oil content of the strains under study, it was interesting to evaluate its effect on the fatty acid profile (Figure 9). The strains producing the highest amounts of fat were grown in an oleogenic medium with the addition of the most common carbon sources (glucose, glycerol, molasses, xylose). In addition, the fatty acid profile was evaluated when ammonium sulfate was replaced with urea.

According to the data obtained, we observe a number of patterns. First, when the carbon source is changed from glucose to another for most strains (except WCh and AV2), a decrease in palmitic acid content is characteristic. We observe a similar pattern for oleic acid, with the exception of strain AV2. As expected, when stearic acid decreases, the content of oleic and linolenic acids increases. When the carbon or nitrogen source is replaced, the relative palmitic acid content varies within 10%, except for the Sil strain, where replacing glucose with glycerol led to a significant (up to 15%) decrease in fatty acid content and a comparable 14% increase in oleic acid.

Another interesting observation is that for most strains (except Sil and WCh), the maximum percentage of stearic fatty acid is achieved when cultivated on glycerol or xylose. The variation in the presence of stearic acid is due to changes in the ratio of unsaturated fatty acids.

### 2.5. Lipidome Analysis of Oleogenic Strains Grown on Different Carbon Sources

**Figure 10:**
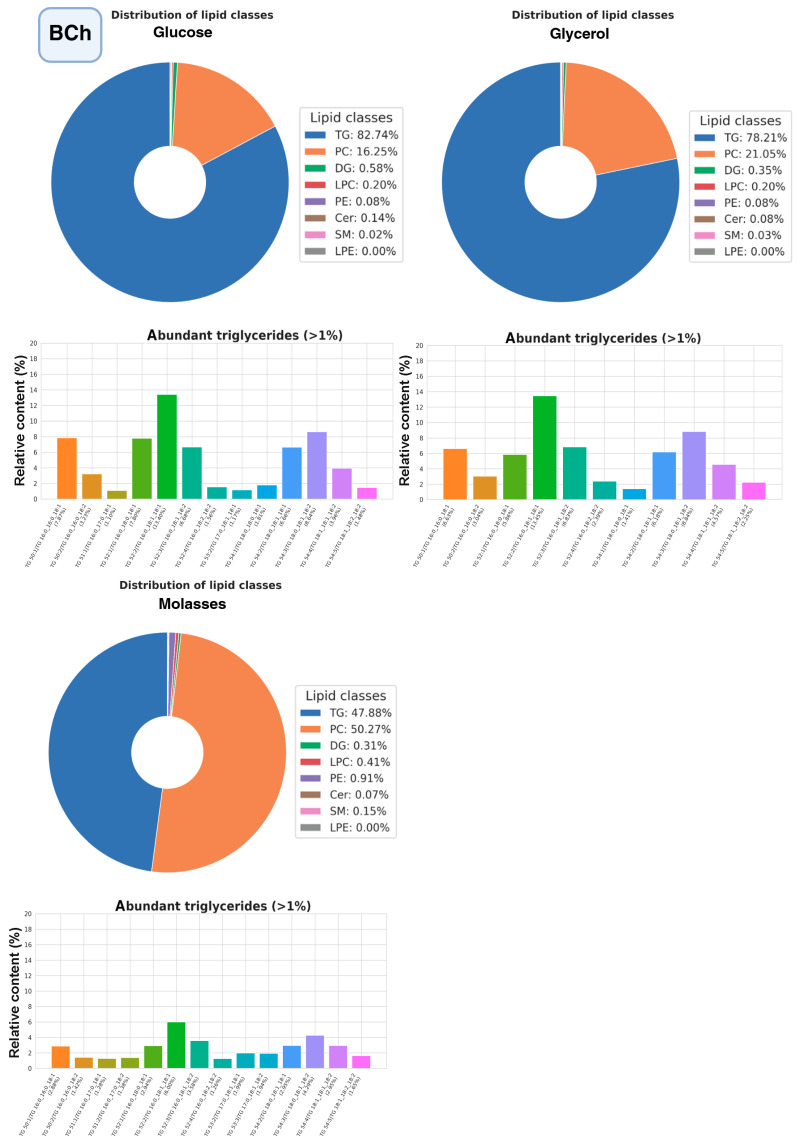
Structure of the lipidome of the BCh strain. Pie chart of the relative content of the 8 major lipid classes. Abbreviations: TG: triacylglycerols; PC: phosphatidylcholines and their ether-linked analogs; DG: diacylglycerols; LPC: lysophosphatidylcholines; PE: phosphatidylethanolamines; Cer: ceramides and related sphingolipids; SM: sphingomyelins; LPE: lysophosphatidylethanolamine. Bar graphs represent the relative abundance of individual TGAs.

**Figure 11:**
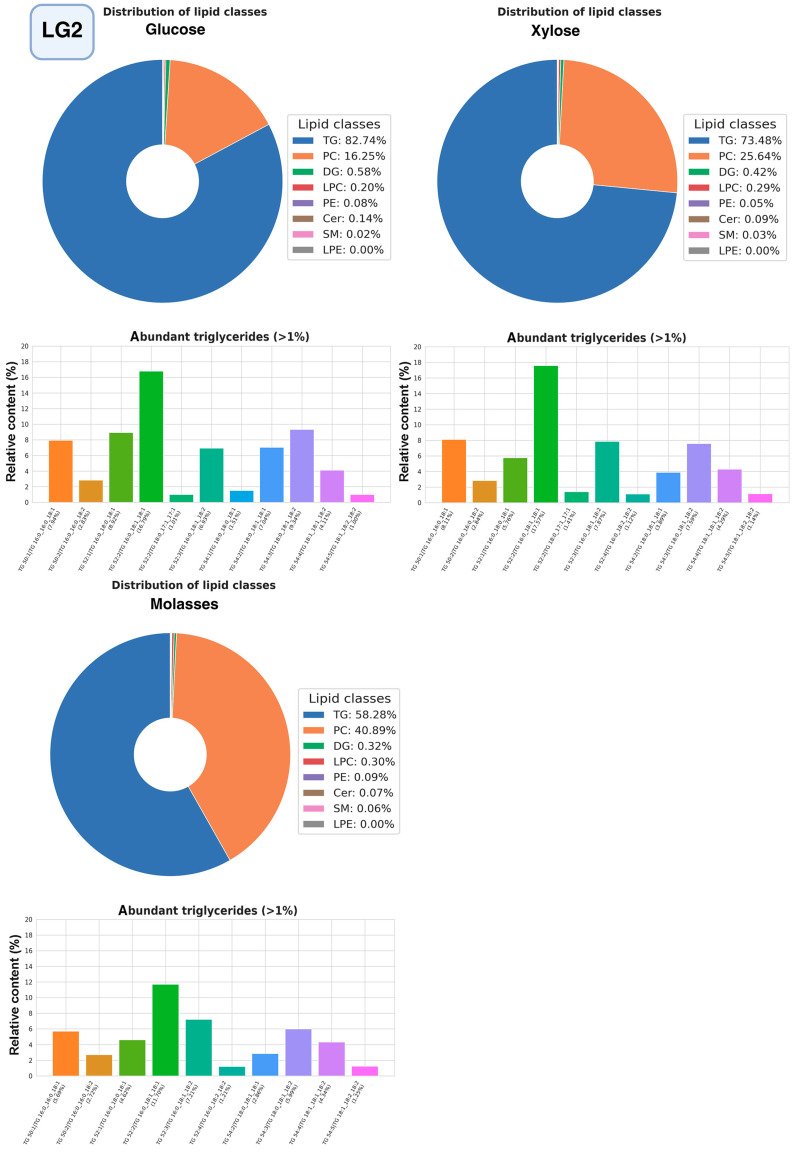
Structure of the lipidome of strain LG2. Pie chart of the relative content of the 8 major lipid classes. Abbreviations: TG: triacylglycerols; PC: phosphatidylcholines and their ether-linked analogs; DG: diacylglycerols; LPC: lysophosphatidylcholines; PE: phosphatidylethanolamines; Cer: ceramides and related sphingolipids; SM: sphingomyelins; LPE: lysophosphatidylethanolamine. Bar graphs represent the relative abundance of individual TGAs.

**Figure 12:**
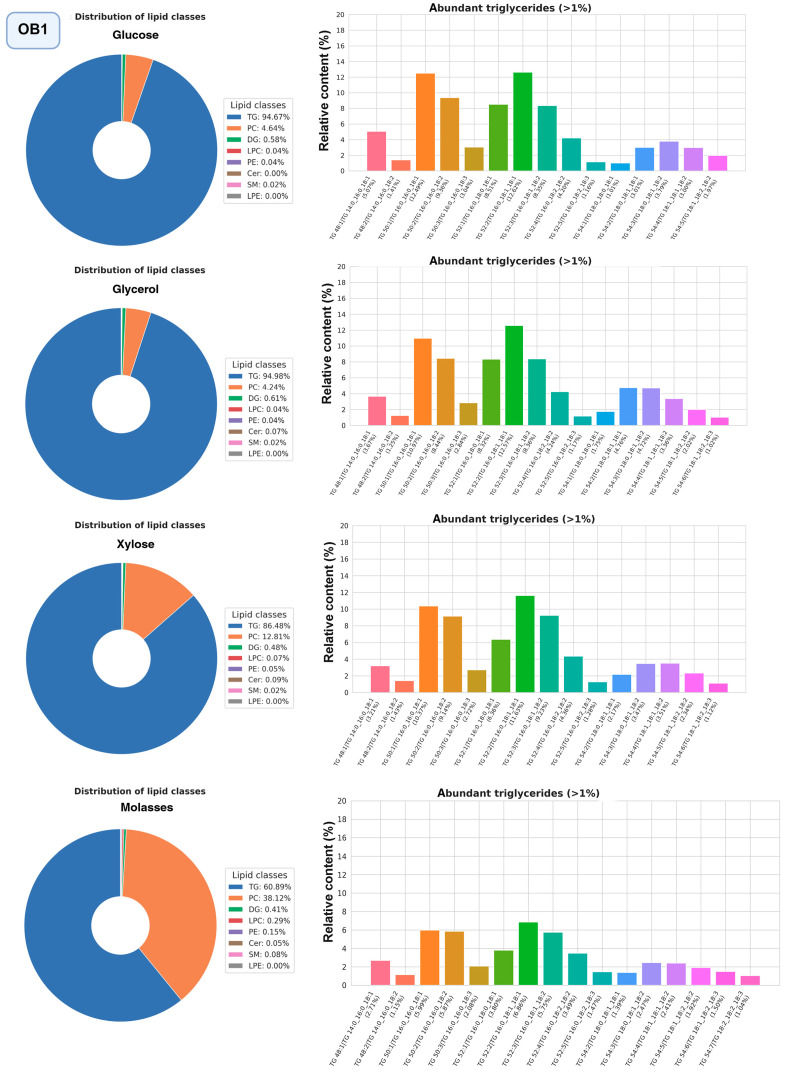
Structure of the lipidome of strain OB1. Pie chart of the relative content of the 8 major lipid classes. Abbreviations: TG: triacylglycerols; PC: phosphatidylcholines and their ether-linked analogs; DG: diacylglycerols; LPC: lysophosphatidylcholines; PE: phosphatidylethanolamines; Cer: ceramides and related sphingolipids; SM: sphingomyelins; LPE: lysophosphatidylethanolamine. Bar graphs represent the relative abundance of individual TGAs.

The most promising strains were selected for studying the composition of lipidomes, including analysis of triglyceride composition depending on the carbon source (glucose, glycerol, or xylose, as well as molasses) (Figure 10, Figure 11 and Figure 12, a more detailed composition of yeast lipidomes is presented in Table S5). Strain BCh produces the highest dry biomass on the oleogenic medium studied among all the strains studied (Figure 6). Strain OB1 accumulates the highest amount of lipid per dry biomass (Figure 6). Strain LG2, having a fairly high oil content, is capable of actively metabolizing all alternative carbon sources (Figure 8).

According to the data obtained (Figure 10), phosphatidylcholine and triglycerides dominate the lipidome structure of the BCh strain, while the content of other lipid groups does not change significantly. The highest amount of triglycerides accumulates when the strain is grown on glucose and the lowest when grown on molasses. Regardless of the carbon source, triglycerides with unsaturated oleic and linoleic fatty acids, such as POO (Palmitate:Oleate:Oleate), POL (Palmitate:Oleate:Linoleate), SOL (Stearate:Oleate:Linoleate), SOO (Stearate:Oleate:Oleate), and PPO (Palmitate:Palmitate:Oleate) predominate among triglycerides.

In strain LG2, as in strain BCh, phosphatidylcholine and triglycerides dominate the lipidome structure (Figure 11), with a relatively constant composition of other lipid groups. The largest amount of triglycerides accumulates when the strain is grown on glucose and the smallest when grown on molasses. Triglycerides with unsaturated fatty acids oleic and linoleic, POO, POL, SOL, SOO, and PPO.

In strain OB1, as in LG2 and BCh, phosphatidylcholine and triglycerides dominate the lipidome structure (Figure 12). It should be noted that, unlike LG2 and BCh, the proportion of triglycerides is higher in OB1. When OB1 is grown on molasses, a significant increase in phosphatidylcholine content (40–50% of the total amount of lipids) is also observed. We suggest that the very high content of free choline (0.9 g per 1 kg [51] in the beet molasses we used) stimulates the biosynthesis of phosphatidylcholine via the CDP-choline pathway [52]. Another difference is observed in the composition of triglycerides: in OB1, triglycerides with saturated fatty acids, such as POS, PPL, and POP, begin to predominate.

### 2.6. Comparison of the Percentage Content of Fatty Acids in Common Vegetable Oils with That in Promising Strains of Oleogenic Yeast Under Study

Next, we compared the fatty acid content of the most promising oleogenic strains with vegetable oils that are in demand in the economy. The data are presented in Table 1.

The LG2 strain accumulates the highest amount of palmitic acid (C16:0), which makes its oil more similar to palm oil. The BCh strain, grown on urea as a nitrogen source, exhibits a fatty acid profile similar to soybean oil. LG2 grown on xylose, OB1 grown on urea, and BCh grown on glycerol produce oil enriched with saturated fatty acids, making their oil more similar to cocoa butter. It should be noted that the oil from none of the strains matches all the fatty acids in the corresponding vegetable oil, so further genetic and metabolic engineering is required to create producers.

## 3. Discussion

Model strains of oleogenic yeast have proven themselves as subjects for the development of biotechnological solutions for the production of fatty acids and oils [56]; however, screening for new strains can facilitate the identification of yeasts that are closer (in terms of fatty acid and TG accumulation profile) to the target product expected to be obtained industrially [29]. As part of this study, we searched for and screened a large number of potentially oleogenic yeasts, from which we selected those that most closely meet the requirements of the microbiological industry.

Among the 100 strains isolated from various sources, 31 yeast strains have the ability to accumulate more than 20% of the mass fraction of oil from biomass. However, only a few strains can be considered productive enough to meet the basic demands of the industry. It is interesting to note that a significant proportion of oleogenic strains (17 out of 31) were isolated from the surface of fruits; oil yeast strains have previously been found in similar sources [57,58,59], including a rapid screening method based on the use of the fluorescent dye Rhodamine B on microorganisms isolated from soil and food waste [60]. In addition, oleaginous yeast is often obtained from food products [61,62], in our case from wine and beer cheeses, condensed milk, sour cream, and acidophilin—but in significantly smaller quantities, apparently due to the heat treatment of food raw materials or biological competition with starter bacteria. It is interesting to note that oleogenic strains were also found in silage, which may be related to the peculiarities of the silage procedure [63], which is also based on the fermentation of plant products.

One of the significant problems in the stream production of yeast isolates for the selection of promising strains remains the low accuracy of routine identification using MALDI-TOF. In our case, using this method, it was possible to establish the genus for only about 50% of the strains. Approximately the same percentage of identified strains remains when considering only oleogenic strains (14 identified out of 31 strains). Despite the possibility of using this tool to identify microorganisms, including in food research [64], regular database updates are required for comfortable operation [37]. In this regard, sequencing specific PCR products is undoubtedly preferable and provides greater reliability, but in the case of routine high-throughput screening, it can be labor-intensive.

Interestingly, different isolates of the same species (using *Debaryomyces hansenii* as an example) are characterized by different lipid accumulation: strains WCh, BCh, and NN17 accumulate more than 20% of lipids from biomass, unlike strains Qsab and Ban1, which do not float in glycerol (Table S2). At the same time, oleogenic strains within the same species are characterized by different fatty acid composition profiles: for example, the levels of C18:0 and C18:1 differ in strains WCh and BCh, with strain BCh accumulating more C18:0 (25.02% vs. 18.82%), while the C18:1 content is higher in strain WCh (42.63% vs. 35.51%), which can be explained by the different metabolism of the strains. In general, oleogenic yeast strains, especially those with biotechnological potential in terms of fatty acid and TG profiles, have relatively low species diversity, which is consistent with the previously expressed position on the difficulties of introducing natural strains into the production cycle [29]. However, even with the low species diversity of the strains we studied, they demonstrate different physiological and biochemical parameters, which justifies both search and screening studies. The results of the search for new strains may be of particular interest in the context of studying the physiology of individual findings: such as strains that grow without a source of nitrogen or carbon, as well as those strains that differ sharply from other yeasts in terms of metabolism.

The main pattern we observe when evaluating the fatty acid profile of yeast is a high content of stearic and palmitic acids; this is also characteristic of other yeasts, for example, some strains of the traditional lipid producer *Y. lipolytica* [65]. We believe that the lipid-producing strains LG2, OB1, and Bch that we have discovered and characterized have great potential for further use in the food industry. It is clear that the quality of the oil extracted from them is not sufficient to serve as an analogue of one or another edible oil, so it is necessary to improve the strains using genetic and metabolic engineering methods. The relatively high palmitic acid content characteristic of the studied strains is observed in such edible vegetable oils as palm (42–45%), cacao (24–33%), olive (10–13%), corn (9–12%), coconut (9–11%), and peanut (8–10%) oils, [3,53,55,66]. Depending on the carbon source, the strains LG2, OB1, and Bch can accumulate significant amounts of stearic acid and smaller amounts of oleic acid, which brings the oil extracted from them closer in composition to shea and cocoa butter (Table 1) [55,67]. However, many vegetable oils (flaxseed, sunflower, Rapeseed, Shea butter) have a low percentage of saturated palmitic and stearic acids (no more than 8%) [54,68]. Metabolic engineering approaches can be used to create yeast oil producers with a higher degree of fatty acid unsaturation. Oleic and palmitoleic acids are produced from stearic and palmitic acids, respectively, with the participation of the enzyme stearoyl-CoA 9-desaturase (or acyl-CoA 9-desaturase) [69] encoded by the *OLE1* gene [70,71]. Accordingly, overexpression of the *OLE1* gene will lead to an increase in the proportion of unsaturated fatty acids. Molecular tools for genetic engineering and genome editing of *Debaryomyces* [72,73,74], and for *Rhodotorula* [75,76], which makes the discussed modifications of the Bch and OB1 strains of potential producer strains quite realistic. However, no genetic engineering and genome editing tools have yet been described in the literature for the genus *Vanrija*. In addition to modifying the expression of key fatty acid metabolism genes, carbon and nitrogen sources strongly influence fatty acid composition. Thus, according to our data, the use of molasses or urea can reduce the stearic acid content (Figure 9). In addition to altering the activity of enzymes responsible for fatty acid biosynthesis, other metabolic engineering approaches that affect fatty acid composition include altering the activity of enzymes responsible for fatty acid breakdown (beta-oxidation pathway) and triglyceride synthesis [77].

Taken together, these observations justify both the need to search for new strains of oleogenic yeast from various sources and the importance of conducting inexpensive studies to describe their basic physiological characteristics. This set of experiments—determining oil content, the ability to metabolize various sources of carbon and nitrogen, and determining the fatty acid profile—is a preliminary screening for further detailed examination of the metabolic characteristics of the most intriguing strains. Similar approaches have been proposed for other industries, such as winemaking and biodiesel production [78,79,80], up to the scaling stage [81]. We demonstrate the feasibility of this approach using three strains (BCh, LG2, and OB1), which can be used as yeast producers of oil analogues for the food industry in the further development of metabolic engineering approaches. It should be noted that the fat content of the yeast strains studied, the amount of oil they produce, and its composition were obtained using laboratory methods. Certain problems can be expected when scaling up the quantity and quality of oil produced to industrial levels, the solution to which will most likely lie in optimizing the composition of the nutrient medium and the conditions of controlled fermentation.

The limitations of this study are that the standard oleogenic medium used in the work may not be optimal for the best strains identified, and systematic selection of cultivation conditions and oleogenic medium composition is necessary to increase the content of triglycerides specific to edible oils. In addition, the oleogenic medium used may not be optimal for the production of biomass of the studied strains; therefore, it is necessary to optimize the cultivation conditions and the composition of the nutrient medium to ensure the production of increased biomass of the studied strains. The oil extraction methods used in the work are not compatible with the further use of the extracted oil for food purposes, therefore it is necessary to select conditions and modify methods for extracting fat for food purposes.

## 4. Materials and Methods

### 4.1. Sources of Oleogenic Strains, Isolation, and Cultivation

In 2023–2025, we studied and analyzed endophytic yeasts from the internal tissues of 41 agricultural crops (fruits, vegetables) obtained through trade networks in the Moscow region, as well as natural and biotechnologically valuable substrates such as soil, ash sawdust, corn, and grass stover. The list of strains obtained and their origin are given in Table S6. Epiphytic yeast isolates from the surface of fruits were obtained as follows. The fruits selected as sources were rolled over the surface of Petri dishes with a solid nutrient medium of yeast peptone dextrose (YPD) agar (20 g/L glucose, 20 g/L peptone, 10 g/L yeast extract, 20 g/L agar) with the addition of levofloxacin and ciprofloxacin. The dishes were then incubated at +4 °C until yeast colonies appeared. Individual colonies were subcultured several times until pure yeast cultures were obtained. Purified yeast strains were cryopreserved in 10% (*v*/*v*) glycerol in water solution at −80 °C.

### 4.2. Physiological Test of Yeasts: Determination of the Ability to Grow on Different Carbon Sources, Growth Curves, and Biomass Accumulation

Investigated strains were characterized morphologically and physiologically using standard methods on standard solid and liquid media described in Kurtzman et al. [82]. Briefly, physiological tests for the ability to assimilate various carbon and nitrogen sources, as well as growth at different temperatures and under osmotic stress, were performed in duplicate on YNB-based minimal media without amino acids (https://www.sigmaaldrich.com/RU/ru/product/sigma/y0626 (accessed on 09 November 2025)) for the assimilation of various carbon sources and YCB (https://www.sigmaaldrich.com/RU/ru/product/sigma/y3627 (accessed on 09 November 2025)) for the assimilation of various nitrogen sources. Inoculations on YNB-based minimal media without amino acids and YCB without added carbon and nitrogen sources were used as negative controls, respectively. YNB medium supplemented with glucose, on which inoculations from the strain collection were incubated at room temperature (25 °C), was used as a positive control. The ability to assimilate various sources was assessed 7 days after inoculation on assimilation media. Assimilation was assessed using the following gradation:

No growth (−)—no growth

Low (− +)—single colonies

Medium (− +)—continuous biomass growth, but significantly less than in the positive control

High (+)—biomass growth comparable to the positive control or more abundant.

These experiments were conducted in two biological replicates to exclude erroneous results. If contradictions were found, the experiments were repeated twice more.

Growth rate measurement of oleogenic strains was performed as described in Kashko et al. [83] with minor modifications. Briefly, yeast strains were grown on various carbon/nitrogen sources in a Thermofisher Multiscan FC plate reader (Thermo Fisher Scientific, Waltham, MA, USA). For this purpose, overnight cultures of the strains under study were inoculated into PBS, diluted to a density of OD600 = 0.333, and then 30 μL of the resulting cell solution in buffer was added to wells containing 70 μL of YPD (positive control), oleogenic medium based on various carbon and nitrogen sources to a final volume of 100 μL with an initial cell density of OD600 = 0.1. As a negative control, the test media without yeast cultures were applied to the wells of the plate. Measurements were performed at room temperature (25 °C) with regular shaking and recording of the optical density of the culture every 25 min for 42 h using a 620 nm light filter. The growth rate in the exponential phase (µ) was calculated using a custom script written in R (version 4.4.0.) and deposited on GitHub (https://github.com/Knorre-lab/GrowthCurves, accessed 19 September 2025).

### 4.3. Determination of the Lipid Content of Yeast Strains

To determine the relative percentage of oil, the yeast strain was sown on PDB-agar (4 g/L potato extract, 20 g/L glucose, 20 g/L agar) to separate colonies and grown for 5–7 days at room temperature. After that, the large biomass of the colony was scraped off with a loop and carefully placed in a glycerol solution, trying not to resuspend it. The experiment was repeated at least twice. If the biomass did not sink in both experiments, the strain was considered fatty with a baseline lipid accumulation level of more than 20% of dry weight. Typically, the fat content of the strains that floated in 20% glycerin were at least 25–30%.

### 4.4. Gravimetric Determination of the Mass Fraction of Lipids

To determine the level of lipid accumulation, yeast biomass was grown for 5 days at 200 rpm and 25 °C in 100 mL of medium A on different carbon sources (glucose, xylose, glycerol, acetate, molasses) and nitrogen (ammonium sulfate or urea) (carbon source 30 g, yeast extract 1.5 g, ammonium sulfate 0.6 g (urea 0.3 g), KH_2_PO_4_ 7 g, Na_2_HPO_4_·12H_2_O 5 g, MgSO_4_·7H_2_O 1.5 g, FeCl_3_·7H_2_O 0.08 g, ZnCl_2_·6H_2_O 0.012 g, CaCl_2_ 0.1 g, MnSO_4_·5H_2_O 0.1 mg, CuSO_4_·5H_2_O 0.1 mg, CoCl_2_·6H_2_O 0.1 mg) in 500 mL Erlenmeyer flasks with baffles (Baffled flask), maintaining a ratio of medium volume to flask volume of 1:5. The inoculum was pre-grown from a colony or museum on 3 mL of YPD medium. For sowing, 1 mL of overnight culture was taken per flask.

The yeast biomass was precipitated by centrifugation at 3000× *g* for 2 min at room temperature in pre-weighed 50 mL centrifuge tubes and dried at 105 °C overnight. The resulting dry biomass was ground. A 100 mg sample was taken in a 2 mL homogenizer tube. 0.25 mL of ceramic beads (d = 0.5 mm) and 3 beads (d = 4 mm) were added to each tube. Then, Folch’s reagent chloroform:methanol:water was added in a ratio of 2:1:0.8 (by volume) up to the 1.5 mL mark. The tubes were cooled on ice, placed in a Precellys 24 homogenizer (Montigny-le-Bretonneux, France) and homogenized three times at 6800 rpm for 30 s, cooling the tubes on ice after each cycle. The liquid phase was collected from the tube and transferred to a 15 mL centrifuge tube. The residue in the homogenization tube was washed three times with 1.5 mL of Folch’s reagent and transferred to a 15 mL centrifuge tube to a final volume of 6 mL. 1.2 mL of 0.9% NaCl was added to the resulting volume and gently mixed by shaking. To separate the phases, the tubes were centrifuged at 3200× *g* for 2 min. At the end of centrifugation, the formation of three phases was visually observed: the upper phase was aqueous, the middle phase consisted of cell debris, and the lower phase was chloroform containing lipids. The chloroform phase was carefully transferred to pre-weighed tubes, then weighed again. The chloroform was evaporated and the tubes containing lipid residues at the bottom were weighed again. The proportion of lipids in the dry biomass of yeast was calculated using the following formula.

$$LC\left(\%\right)=\left[\frac{m_{1}-m_{t}}{3}\times V_{c}\right]\times \frac{1}{m_{DCB}}\times 100,$$

where

*m_l_*—mass of the lipid sample

*m_t_*—tare weight (test tube)

*V_c_*—total volume of the chloroform phase

*m_DCB_*_—_mass of dry yeast biomass sample

LC(%)—mass fraction of lipids, %

In the pool of strains that passed the oleogenicity test to verify the rapid method of screening for oleogenicity, the mass fraction of lipids was determined, as well as the composition of fatty acids was determined. This check was carried out in a single repeat. According to the results of this experiment, the strains capable of accumulating the largest amount of lipids and yielding the largest biomass were selected for further studies on the assimilation of various carbon and nitrogen sources, as well as studying changes in the profile of fatty acids. Experiments with strains that showed the highest productivity were carried out in 3 biological repeats.

Standard deviations were calculated and confidence intervals were constructed for the rates of assimilation of various substrates. Also, to assess the significance of the results obtained and compare the differences in growth rates on different substrates, the data obtained were processed in one direction using Tukey’s multiple comparison testing, which is displayed on the graphs by ranges of values, the *p* value is adjusted: ****—<0.0001; ***—0.0001< *p* < 0.001; **—0.001 < *p* < 0.01; *—0.01 < *p* < 0.1. The initial data without processing can be found in Table S4, they are presented for the best strains.

### 4.5. Determination of Fatty Acid Profile Using GC-MS

Fatty acid methyl esters (FAMEs) were prepared as described previously with some modification [84]. The FAMEs analysis scheme is shown in Figure S1. Briefly, yeast pellets were washed from cultured media and were resuspended in a 1.5 mL Folch’s reagent with 200 µL glass beads (Sigma-Aldrich, St. Louis, MO USA) [85]. The tubes were placed in a Precellys 24 homogenizer (Bertin Technologies, Montigny-le-Bretonneux, France) at 6800 rpm for 30 s and cooled on ice after each homogenization cycle. After liquid was transferred to a 15 mL tube, washing the homogenization tube three times with Folch’s reagent (1.5 mL) to a final transferred volume of 6 mL. Next, 1.2 mL of 0.9% NaCl was added and gently stirred gently on a vortex and then centrifuged at 3200× *g* for 2 min until the phases were separated. As a result, three phases were formed: upper—methanol phase, middle—from cell residues, lower—chloroform phase containing lipids. After transferring the lower chloroform fraction into vials for GC-MS, by carefully piercing the lower layer, the extract was evaporated stepwise using a rotary evaporator. Then HCl-MeOH solution (500 µL) was added to dried samples and heated at 80 °C for 1 h. Afterwards, FAMEs were extracted by mixing with 500 µL hexane for 10 min. The top phase was transferred into a new vial and used for analysis through GC-MS.

GC-MS analyses were carried out using Agilent 7820A (Agilent technologies, Santa Clara, CA, USA) gas chromatograph with a Maestro MS detector (Interlab, Moscow, Russia) with 25 m × 0.2 mm × 1.12 µm capillary column DB-624 (Agilent technologies, Santa Clara, CA, USA). The injection volume was 1 μL, with a split ratio of 10:1 splitting gas-carrier. Injector and interface temperatures were 250 and 280 °C, respectively. The temperature program for the column started at 125 °C for 1 min, and then rose to 200 at 20 °C/min, and then to 250 °C at 3 °C/min; the end temperature was held for 10 min. Electron impact (EI) spectra were obtained under 70 eV ionization voltage and 150 °C source temperature. Registration of spectra was performed through a full scan at 40–550 Th mass range. Post-run analysis was performed with the following software: Agilent Mass Hunter Unknown Analysis B.07.02.1938 (Agilent Technologies, Santa Clara, CA, USA), Enhanced Data Analysis F.01.01.2317 (Agilent Technologies, Santa Clara, CA, USA), NIST MS Search 2.2 (NIST, Gaithersburg, MD, USA), and Microsoft Excel LTSC MSO (16.0.14332.20579) (Microsoft, Redmond, WA, USA).

Internal standard, calibration strategy and quality control procedures. Tridecanoic acid (C13:0) is used as an internal standard (IS) in all samples, as this acid is not present in yeast. To construct the calibration curve, five different concentrations ranging from 1 to 10 μg/mL were prepared and analyzed based on [86]. A standard solution of fatty acids (c:12, c:13, c:17) was used as a quality control, in which each component was identified and quantified five times using a known amount of tridecanoic acid. Each sample was washed with hexane in the column before the next step. FAME standards (Sigma-Aldrich, St. Louis, MO, USA) and calibration mixtures were dissolved in n-hexane and stored at +4 °C.

### 4.6. Yeast Taxonomic Identification by MALDI-TOF Mass Spectrometry Analysis and ITS Site Sequencing

All strains were grown under identical conditions: YPD, 25 °C, 48 h. Sample preparation for yeast identification by the MALDI-TOF MS method was carried out according to Sauer et al. [87] with minor modifications. The biomass of microorganisms was collected with a 1 μL plastic microbiological loop and resuspended in 300 µL of deionized water. To the suspension was added 900 µL of 96% ethanol, the resulting mixture was mixed thoroughly and centrifuged at 18,894× *g* for 2 min. To the air-dried precipitate, 5 to 40 µL of 70% formic acid was added (depending on the volume of the precipitate) and an equal volume of acetonitrile. The resulting mixture was centrifuged at 18,894× *g* for 2 min. The supernatant containing the protein extract was used in mass spectrometric analysis.

A 384-well steel target plate (Bruker Daltonics, Bremen, Germany) was covered with 1 µL of supernatant and dried at room temperature. On the surface of the dried extract, 1 μL of matrix solution was applied: saturated solution of α-cyano-4-hydroxycinnamic acid (25 mg/mL) (Sigma Aldrich, St. Louis, MO USA) containing 50% acetonitrile and 2.5% trifluoroacetic acid (Panreac, Chicago, IL, USA), which was also dried at room temperature. Mass spectrometric analysis was performed on an UltrafleXtreme mass spectrometer (Bruker Daltonics, Bremen, Germany) equipped with a Nd:Yag laser (355 nm) in linear mode. The positively charged ions in the range from 2000 to 20,000 Th with the following ion source settings: voltage at IS1 20 kV, at IS2 19 kV, at lenses (parameter “Lens”) 4.5 kV, detector supply voltage 2885 V, gain 12.6.

Spectra were taken in automatic mode using the Flex Control program (v.3.4, build 135). The points of laser firing on the target were chosen randomly. 1200 spectra from 200 firing points at a laser frequency of 2 kHz.

Spectral libraries of each sample were obtained by analyzing biological triplicates in eight technical repeats. The calibration standard and positive control was Escherichia coli DH5α protein extract with additional proteins (RNase A [M + H]+ 13,683.2 Da, myoglobin [M + H]+ 16,952.3 Da) (cat. no. 255343, Bruker Daltonics, Germany).

The spectra obtained were processed using the MALDI Biotyper Compass Explorer 4.1 software package (Bruker Daltonics, Bremen, Germany) with the Biotyper Preprocessing Standard Method. The processed spectra of the samples were compared with the reference database of characteristic spectral profiles, which included 12,641 records. The results of the characteristic profile search were expressed as the logarithm of the values. Values below 1.699 corresponded to an unreliable identification of genus; 1.700–1.999 corresponded to a reliable identification of genus and possibly species; 2.000–2.299 corresponded to a reliable identification of genus and with a high probability of species; and finally, values of 2.300–3.000 corresponded to a reliable identification to species.

The yeast strains (pure cultures) were molecularly identified using the ITS rDNA region as a universal DNA barcode for fungi [88]. The nuclear ribosomal ITS1-5.8S-ITS2 region was amplified and sequenced using ITS5 primer. The criteria described in [89] were used to separate the yeast species. DNA isolation and PCR were performed according to the procedure described previously [90,91]. DNA sequencing was performed using the Big Dye Terminator V3.1 Cycle Sequencing Kit (Applied Biosystems, Waltham, MA, USA) with subsequent analysis of the reaction products on an Applied Biosystems 3130xl Genetic Analyzer at the facilities of Evrogen (Moscow, Russia). For sequencing, the ITS5 primer (5′-GGAAGTAAAAGTCGTAACAAGG) was used [91].

The sequencing data were checked with FastQC: 36 samples passed per-sequence quality control and 6 passed with warnings. Subsequent trimming with Trimmomatic v0.40 (TRAILING:20; MINLEN:50) did not materially alter the per-sequence quality profiles; the 6 flagged samples remained in the Phred 22–27 range.

Sequencing results were labeled according to prior MALDI-TOF identifications, with secondary confirmation by BLASTn (version 2.17.0) against the NCBI nt database. Samples with ≥98% percent identity were identified to species; all others were reported as sp. No sample had a percent identity lower than 95.4%. All sequences were compared to ITS sequences from type material; the closest type matches were used as references in the phylogenetic analysis. Study sequences and references were aligned with MAFFT v7.520 (auto settings).

Phylogenetic analysis was performed in IQ-TREE v2.2.0 with ModelFinder Plus for model selection and 1000 ultrafast bootstrap replicates to assess branch support. Trees were visualized in iTOL.

### 4.7. Panoramic Lipid Analysis Using LC-MS

The schematic diagram of the panoramic analysis of yeast lipidome is presented in Figure S2. To obtain images for lipid analysis, yeast biomass was grown for 5 days at 200 rpm and 25 °C in 100 mL of medium A. Yeast biomass was precipitated by centrifugation at 3000× *g* for 5 min. The cell pellet was washed with clean water. It was dried in a thermostat at 105 °C overnight. The dry yeast biomass was ground by grinding with a pestle in a mortar. 100 mg of ground dry yeast biomass was mixed with 1 mL of chloroform:isopropanol 2:1 extraction solution. The samples were carefully pipetted and then transferred to Eppendorf tubes. The samples were centrifuged at 13,000 rpm for 10 min at room temperature. 800 µL of the suspension was transferred to vials for further analysis.

An Agilent 6546 time-of-flight mass spectrometer with an Agilent Infinity II liquid chromatograph was used for the analysis. Ion source settings: Fragmentor = 125, Sheath Gas Flow = 12, Sheath Gas Temp = 300, Gas Temp = 200, Nebulizer = 50, Oct RF = 750, VCap = 3500 and 2500, for positive ionization mode of the sample. Ion detection was performed in positive ionization mode of the sample in IDA analysis mode in the scanning range of 300–2000 *m*/*z*.

Chromatographic separation of the components of each sample studied was performed in RPLC chromatography mode using a Waters ACQUITY C8 2.1 × 100 mm 1.7 µm chromatographic column: phase A (water:acetonitrile (4:6); 10 mM ammonium formate, 0.2 mM sodium fluoride); phase B (acetonitrile:isopropanol (1:9); 10 mM ammonium formate, 0.2 mM sodium fluoride); sample injection volume 2 µL. Chromatographic gradient: 0 min 10% B; 2 min 30% B; 2.5 min 48% B; 11 min 65% B; 12 min 99% B; 14 min 99% B; 14.1 min 10% B; 16 min 10% B; flow rate 0.55 mL/min, thermostat temperature 45 °C.

The obtained chromatogram data were processed as follows. Yeast lipid extracts were analyzed alongside three procedural blank samples processed in parallel. Prior to quantification, all lipids annotated as “RIKEN” standards were excluded from the dataset. For each lipid species, the signal intensities from the three blank replicates were averaged. Blank-subtracted intensities were then calculated for every lipid in each biological sample by subtracting the corresponding mean blank value; any resulting negative values were set to zero. Relative abundances (%) were subsequently computed for each of the 172 detected lipid species by normalizing individual intensities to the total lipid signal per sample.

For the analysis, an Agilent 6546 time-of-flight mass spectrometer coupled to an Agilent 1290 Infinity II UHPLC system was used. The instrument was calibrated using the manufacturer’s calibration solution (“Agilent ESI-L (Low Concentration) TOF reference mass solution”) and according to the manufacturer’s protocols in positive ionization mode over the *m*/*z* range 50–3200 (a wider range than that used for detection during the analysis). Calibration accuracy during the analytical runs was evaluated based on the exact masses of annotated compounds; the mass error did not exceed 2–7 ppm.

Internal standard. Isotopically labeled internal standards were not used for normalization in this study, because the analytical objective was not to isolate biological production from the workflow, but to quantify the recoverable lipid output per unit biomass under a semi-industrial extraction process. Therefore, all samples were normalized to dry yeast biomass (dry cell weight, DCW).

Lipid species were grouped into the following structural classes for compositional analysis: triacylglycerols (TG), phosphatidylcholines and their ether-linked analogs (PC & EtherPC), diacylglycerols (DG), lysophosphatidylcholines (LPC), phosphatidylethanolamines (PE), ceramides and related sphingolipids (including Cer_HS, Cer_AP, and HexCer_HS), sphingomyelins (SM), and lysophosphatidylethanolamine (LPE). The relative distribution of these classes was visualized for each sample using pie charts.

Furthermore, triacylglycerol species contributing more than 1% or 5% of the total lipidome were identified per sample. Bar plots were generated to display the relative abundances of these abundant TG species, with consistent color mapping across visualizations to facilitate comparison.

## 5. Conclusions

Our study demonstrates the benefits of searching for and researching natural yeast strains both from the point of view of fundamental science and for the creation of future innovative biotechnological solutions in the food industry. As part of this work, we have created a collection of yeasts from various sources, among which we have discovered and characterized in detail three oleogenic strains that biosynthesize oil similar to known edible oils. As a further development of our work, we plan to use genetic and metabolic engineering approaches to increase the production of triglycerides specific to edible oils in order to obtain improved strains that produce edible oil analogues and assess their potential for industrial use.

## Figures and Tables

**Table 1:** Comparison of the fatty acid composition of palm oil with strain LG2, cultivated on glucose; soybean oil with strain BCh cultivated on urea; cacao butter with strains LG2 cultivated on xylose, OB1 cultivated on urea, and BCh cultivated on glycerol. All numerical values are given as percentages.

Fatty Acid	Palm Oil [53]	LG2, Glucose	Soybean Oil [54]	BCh, Urea	Cacao Butter [55]	LG2, Xylose	OB1, Urea	BCh, Glycerol
C14:0	<1	0.19	<1	0.08	<4	0.00	0.03	0.1
C16:0	42–45	33.05	10–14	23.52	24–33	29.80	29.16	25.94
C16:1	<1	1.27	<1	1.78	<4	1.25	0.673	1.8
C18:0	4–8	11.7	4–8	14.95	33–40	24.09	11.47	34.75
C18:1	38–42	45.62	20–23	40.46	26–35	32.06	29.28	24.27
C18:2	9–12	6.04	51–54	13.62	<3	9.90	15.59	9.61
C18:3	<1	0	6–10	2.83	<1	0.44	9.51	1.87

## Data Availability

The original contributions presented in this study are included in the article/Supplementary Material. Further inquiries can be directed to the corresponding author.

## Supplementary Materials

The following supporting information can be downloaded at: https://www.mdpi.com/article/10.3390/ijms27020578/s1.

## Abbreviations

The following abbreviations are used in this manuscript:

DG	diacylglycerol
EtherPC	ether-linked phosphatidylcholine
ITS	Internal transcribed sequence
LPE	lysophosphatidylethanolamine
PC	phosphatidylcholine
PE	phosphatidylethanolamine
TG	triacylglycerols
SM	sphingomyelin
