# Supplementary figures and images for: Characterization of a Collection of Natural Oleogenic Yeasts to Identify Promising Producers of Food Oil Analogues
Source: Int J Mol Sci. 2026 Jan 6;27(2):578. doi: 10.3390/ijms27020578 (PMC12840915; doi:10.3390/ijms27020578)

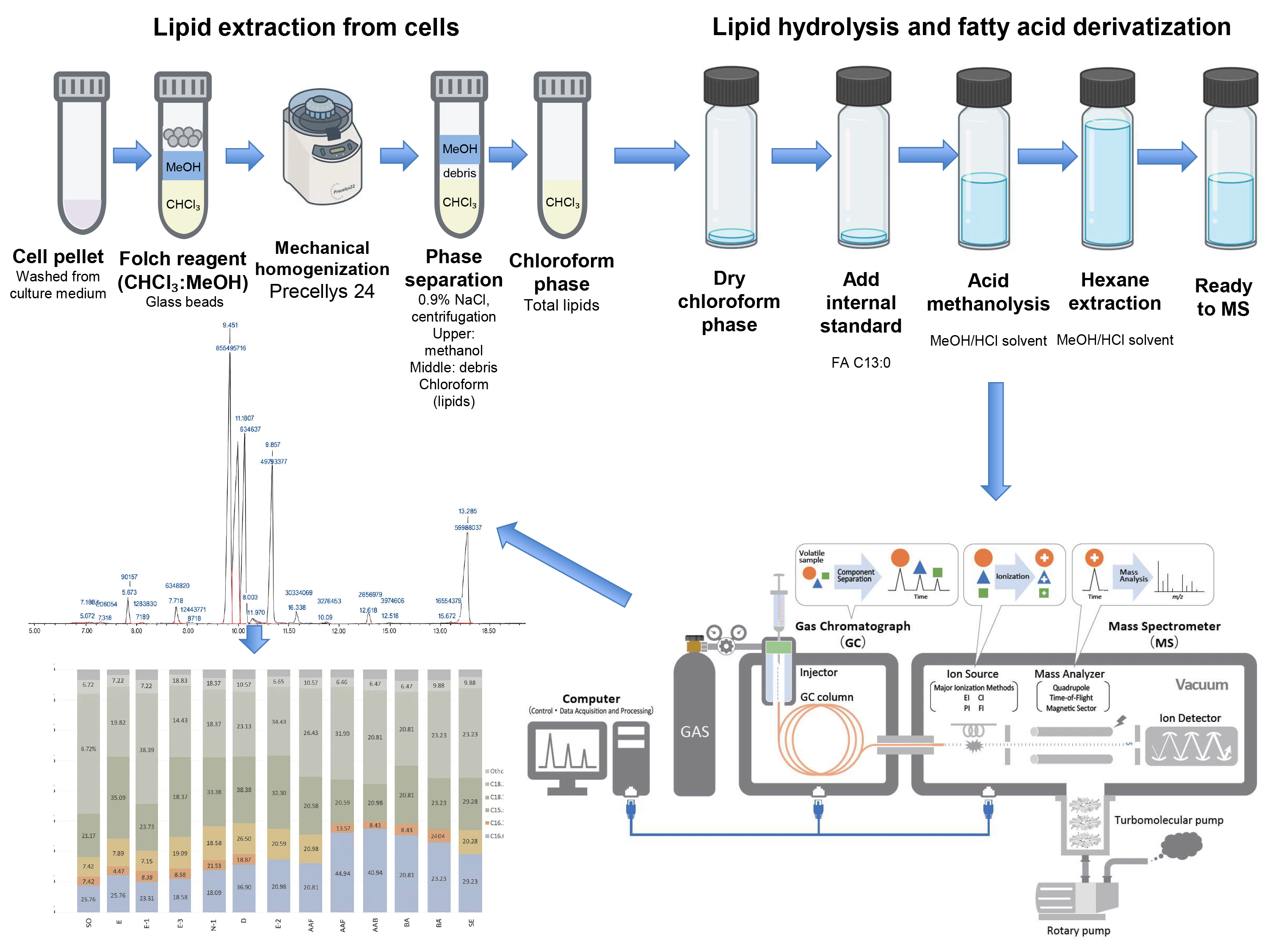

Supplement: Supplementary file 1 [file ijms-27-00578-s001.zip › Figure S1 Scheme of fatty acid profile analysis.tif]

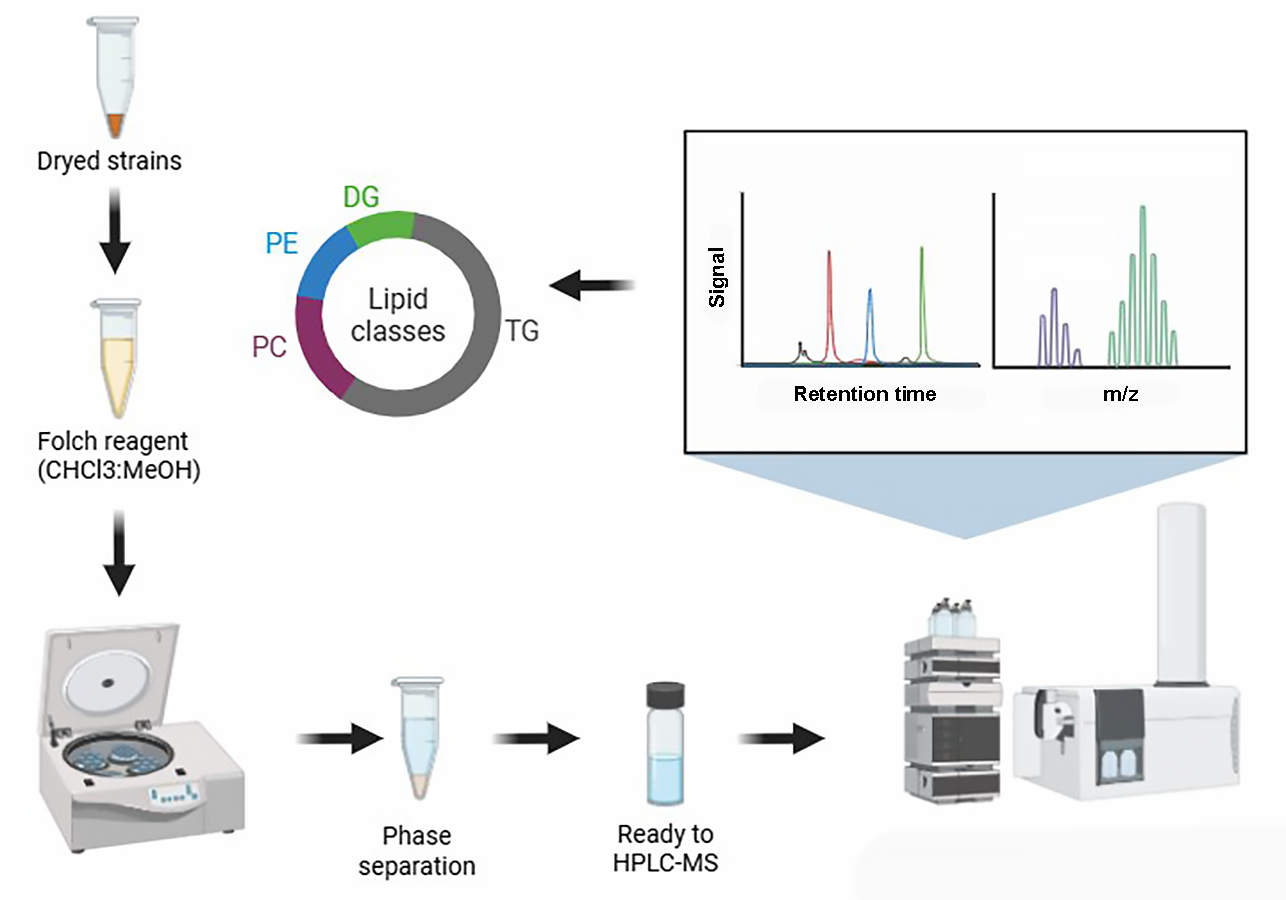

Supplement: Supplementary file 1 [file ijms-27-00578-s001.zip › Figure S2 Schematic representation of lipidome analysis using LC-MS.tif]
